# A fully functionalized metamaterial perfect absorber with simple design and implementation

**DOI:** 10.1038/srep36244

**Published:** 2016-10-26

**Authors:** Sze Ming Fu, Yan Kai Zhong, Ming Hsiang Tu, Bo Ruei Chen, Albert Lin

**Affiliations:** 1 Department of Electronics Engineering, National Chiao-Tung University, 1001 University Road, Hsinchu, Taiwan

## Abstract

Broadband perfect metamaterial absorbers have been drawing significant attention in recent years. A close-to-unity absorption over a broad spectral range is established and this facilitates many photonic applications. A more challenging goal is to construct a broadband absorber with a tailored spectral absorption. The spectral absorption control and spectral shaping are very critical in many applications, such as thermal-photovoltaic, thermal emitters, spectrum imaging system, biomedical and extraterrestrial sensing, and refractive index sensor. In this work, one-dimensional (1D) planar stacking structure is designed to achieve the ultimate goal of a functionalized absorber with a fully tailorable spectral absorption. The lithography and etching process are totally eliminated in this proposed structure, and the fabrication is fully compatible with the regular silicon IC processing. By using ~2 nm ultra-thin metallic layers with a 10-pair (10X) SiO_2_/Si_3_N_4_ integrated dielectric filter, we can achieve decent spectral response shaping. The planar configuration of the ultra-thin-metal metamaterial perfect absorber (MPA) is the key to the easy design/integration of the dielectric filters on top of the MPA. Specifically, band-rejected, high-pass, low-pass and band-pass structure are constructed successfully. Finally, experimental evidence to support our simulation result is also provided, which proves the feasibility of our proposal.

Broadband metamaterial perfect absorbers have been under investigation owing to its versatile applications including thermal photovoltaic (TPV)^1^,^2^,^3^,^4^,^5^,^6^, sensors^7^,^8^, spectral imaging systems^9^, and antenna systems^10^, and cloaking devices^11^. There have been many studies on reaching the goal of a uniform broadband absorption using metamaterial perfect absorber (MPA). Among these efforts, Fang *et al*. proposed the use of hyperbolic metamaterial tapers^12^,^13^. Wen *et al*. propose the use of multiple resonators in proximity for a broadband response^14^. A literature review on the recent progress on MPAs can be found in^15^,^16^,^17^,^18^. Recently, our group proposed ultrathin-metal-based planarized MPA to realize ultra-broadband absorption^19^.

While there has been significant progress in the MPAs in recent years^1^,^2^,^4^,^7^,^15^,^16^,^19^,^20^,^21^,^22^,^23^,^24^,^25^,^26^,^27^,^28^, a more aggressive goal is to attain a total control over the spectral absorption and the cut-off. Therefore, a purely broadband MPA needs to be further tailored to achieve a fully functionalized absorber. A potential way to achieve this is by the construction of a filter on top of the broadband MPA. The main consideration comes from two aspects. The first one is that it is desirable to integrate the filter into the MPA rather than to use a standalone one. The integrated filter can lower the overall cost and also promote the compactness of photonic devices. The second consideration is how to incorporate the filter on top of the MPA while still maintaining its filtering characteristics. The planar ultra-thin metal MPA proposed in our past work^19^ is the key that functional absorbers can be realized since it is very difficult to integrate dielectric filters of any kinds on the top of nano-structured MPAs while still maintaining the same filtering characteristic as the standalone filter.

Here, a one-dimensional (1D) stacking structure is proposed to make absorption spectral control available without any lithography and etching steps. The structure is composed of a filter and an MPA stacked together. The filter is constructed by alternatively depositing 10 pairs (10X) of silicon nitride (Si_3_N_4_) and silicon oxide (SiO_2_) layers, and therefore it is a dielectric filter. Transparency of Si_3_N_4_ and SiO_2_ in the visible (VIS) and the near infrared (NIR) region, i.e. λ = 300 nm to λ = 2600 nm is utilized, and the thickness of each layer will be tuned to spectrally shape the absorption band, achieving a versatile aperiodic design. The perfect absorption is made available by using ~2 nm ultrathin-metal-based planar MPAs^19^. The planar MPA facilitates the design of an integrated filter. In other MPA configurations, their nano-structured nature greatly complicates the design of an integrated filter. Also, the nanostructured MPAs also complicates the fabrication of an integrated filter on top of it. This is because it is hard to control the thin film deposition and step coverage to be totally identical to the calculated one. Figure 1 shows the schematic diagram of the proposed structure of the functionalized MPA implemented with a planar MPA and a planar dielectric filter.

Figure 1 shows the schematics and the concept of the proposed functional absorber. In Fig. 1, λ_1_ and λ_2_ are the wavelengths which are expected to be absorbed in the planar MPA when no filter is added. For the standalone filter spectral characteristic, photons with wavelength λ_1_ can penetrate the dielectric filter, but photons with wavelength λ_2_ will be reflected by the filter. After the dielectric filter is implemented on top of the planar MPA, λ_1_ can still penetrate the filter and then be absorbed by the MPA. On the contrary, λ_2_ will be reflected and thus cannot be absorbed. The reason that we can simply combine the dielectric filter and the MPA is the fully planarized MPA. If we use nanostructured MPAs, the dielectric filter is definitely to be nanostructured, and there will be unwanted diffraction when incident photons go through the filter. In this case, the diffracted power after filter can be more difficult to be fully absorbed by the MPA since the MPA broadband absorption normally degrades with incidence angle. Additionally, the filter characteristic when integrated with MPA changes significantly from its standalone characteristic. Besides, the nanostructured filter plus MPA complicates the design and fabrication, and the process deviation can be very severe, and fabricated devices can be dissimilar to the designed ones.

## Result

### The advanced design for fully functional MPAs

**Figure 2:**
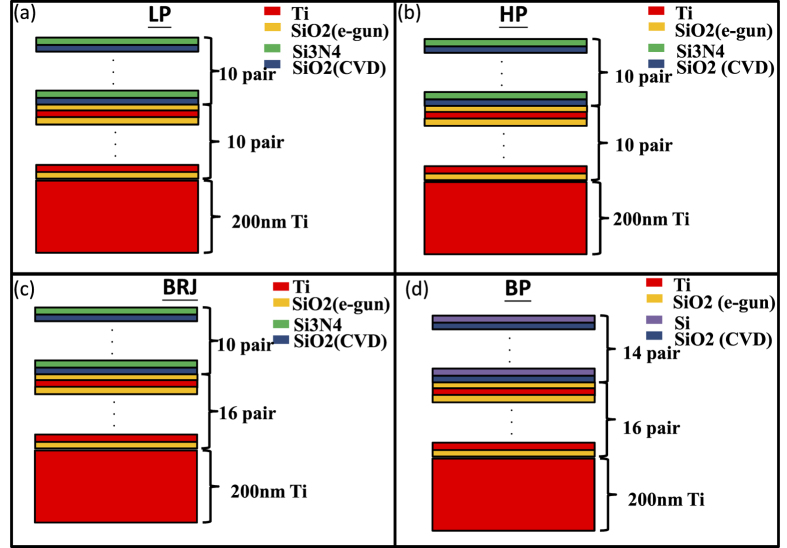
The proposed functional absorber structures. The lower part (Ti/SiO_2_ metal-dielectric alternative layers) acts as a metamaterial perfect absorber (MPA). The upper part (SiO_2_/Si_3_N_4_ or SiO_2_/Si alternative dielectric layers) acts as the filter to select allowed wavelengths. (**a**) Low-pass (LP) structure (**b**) High-pass (HP) structure (**c**) Band-reject (BRJ) structure (**d**) Bandpass (BP) structure.

Figure 2 shows the simulation structure. In Fig. 2, we show the schematic diagram of a 1D filter built on a 1D metamaterial broadband absorber. The broadband absorber is composed of Ti layers (red) and SiO_2_ layers (yellow). The upper part consists of Si_3_N_4_ (green) layers and SiO_2_ (blue) layers. In Fig. 2, SiO_2_ with different colors indicates that they are fabricated by e-gun evaporation or PECVD. The refractive indices by e-gun or PECVD are quite similar based on ellipsometry measurement. The thickness of each layer is different to adjust the spectral response to the desired shape, such as high-pass (HP) or band-reject (BRJ).

### Simulation result

**Figure 3:**
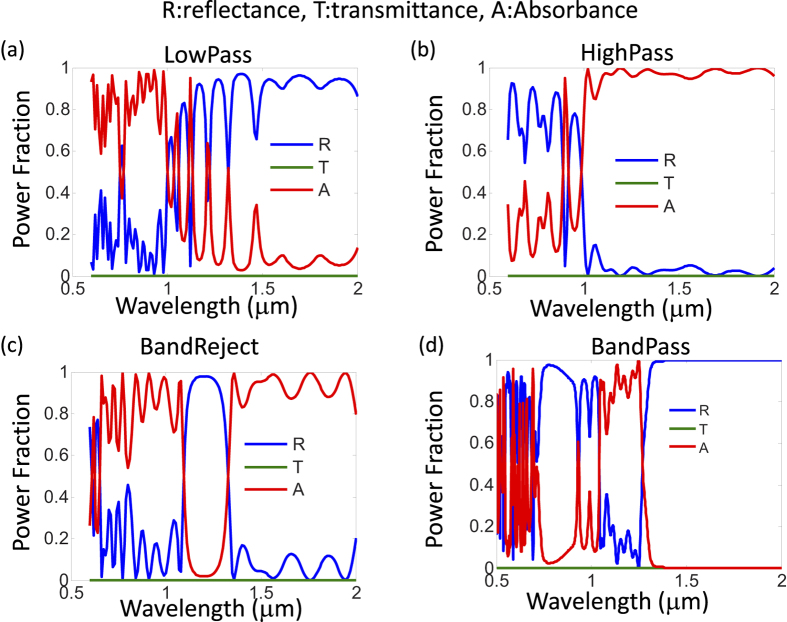
The spectral reflectance (R), transmittance (T), and absorbance (A) for LP, HP, BRJ, and BP functional metamaterial perfect absorbers (MPAs). It should be noted that an improved spectral responses, in terms of cut-off and bandwidth, can be achieved with TiO_2_ or Si as the high index material instead of Si_3_N_4_. Nevertheless, Si_3_N_4_/SiO_2_ system is fully compatible with current silicon IC processing and therefore chosen here.

Figure 3 shows the spectral response for the functional MPAs with low-pass (LP), high-pass (HP), band-pass(BP), band-reject (BRJ) characteristics. The dielectric combination used to construct the dielectric filter is silicon dioxide (SiO_2_)/silicon nitride (Si_3_N_4_) or silicon dioxide (SiO_2_)/silicon (Si). The index contrast is not very large for SiO_2_/Si_3_N_4_ where oxide index is around 1.5 and nitride index is around 2. The advantage of using SiO_2_/Si_3_N_4_ combination is that the transparency of the dielectric filter exists for the entire ultraviolet (UV), visible (VIS), and near-infrared (NIR) spectral range, from λ = 300 nm to λ = 2600 nm. In addition, SiO_2_/Si_3_N_4_ is fully compatible with conventional silicon IC processing and silicon photonic platform. Dielectric filter layers with higher index contrast can further widen the bandwidth and sharpen the spectral absorbance cut-off. Potential alternative candidates to construct the dielectric filter is Si/SiO_2_ and TiO_2_/SiO_2_, where silicon has index around 3.5, and TiO_2_ has index around 2.5. They thus provide higher index contrast. Nevertheless, silicon is not transparent in UV-VIS and therefore can only be used for λ > 1 μm. TiO_2_ is less common in standard silicon IC processing. The aperiodic filter layer thicknesses are optimized by genetic algorithm (GA) and listed in Table 1. The detailed SiO_2_ dielectric spacer thickness (t_SiO2_) and metal thickness (t_Ti_) in MPA can be found in Table 2.

The physics of the functional MPA is that the dielectric filter effectively blocks the photons in the rejection band and transmits the photons in the pass-band. It is worth to mention that the design of an integrated filter with MPA in our case is quite straightforward and not very different from the case of a standalone filter. This is because the MPA is fully planarized. In addition, the photons are absorbed completely by the MPA once they leave the filter stack, and minimal backward reflection from MPA to filter exists. This important point makes the thin-film interference phenomenon in the filter the same for both a standalone filter and an integrated filter on top of an MPA. In the case of nanostructured MPAs, the integration of a filter on MPA becomes complex.

Due to the fundamental optics limitation of the dielectric filters based on wave interference between dielectric multi-layers, the bandwidth of our proposed functional MPA, i.e., the spectral range of the rejection band and the pass-band, cannot be ultra-wide in Fig. 3. Increasing the index contrast in the dielectric filter can somehow increase the bandwidth but may still not be ultra-wideband. In order to achieve ultra-wide bandwidth, absorption type filters can be used on top of the ultrathin-metal planar MPA. The bandwidth of the absorption type filter can be as wide as several micrometers. The absorption type filters are quite different from the dielectric filters in their filtering principle although both of them can achieve spectral selectivity. Taking band-reject (BRJ) filter as an example, the dielectric filter reflects the photons in the rejection band and transmits the photons in the pass band. The wave interference in the multiple dielectric films is utilized to achieve spectral selectivity. On the other hand, absorption (BRJ) filter absorbs the photons in the reject band rather than reflects them. In this regard, absorption filter is not suitable for certain applications such as thermophotovoltaics (TPV) since the photon absorption in the filter itself can generate heat and affect the TPV absorber-emitter temperature. For some other applications such as sensing, the p-n photodiode below the absorption filter generates the photocurrent as the result of environment change, and in this case, the use of an absorption or dielectric filter does not matter since only the photon absorption in the photodiode is counted toward the sensor output signal. The advantage of using absorption filters is their much wider bandwidth compared to the dielectric ones.

**Figure 4:**
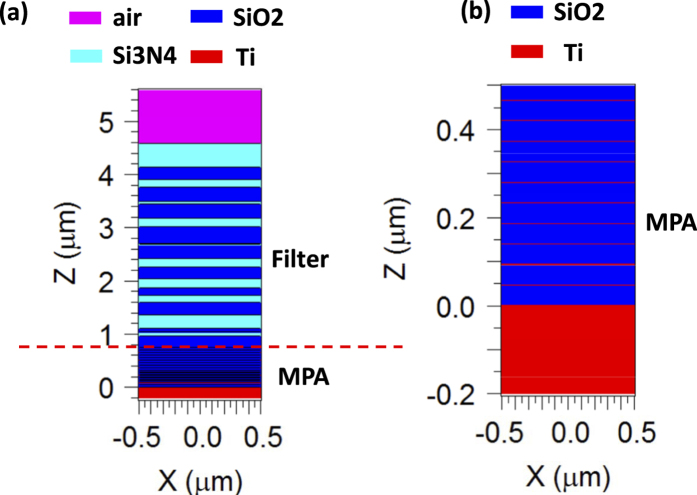
The index profile for the BRJ MPA. (**a**) The index profile of the entire structure using 14 nm resolution in the grid. The ultrathin metal films in the MPA are not resolved since the grid is greater than the metal thickness. (**b**) The fine resolution plot using a 0.5 nm grid to resolve the MPA part solely.

**Figure 5:**
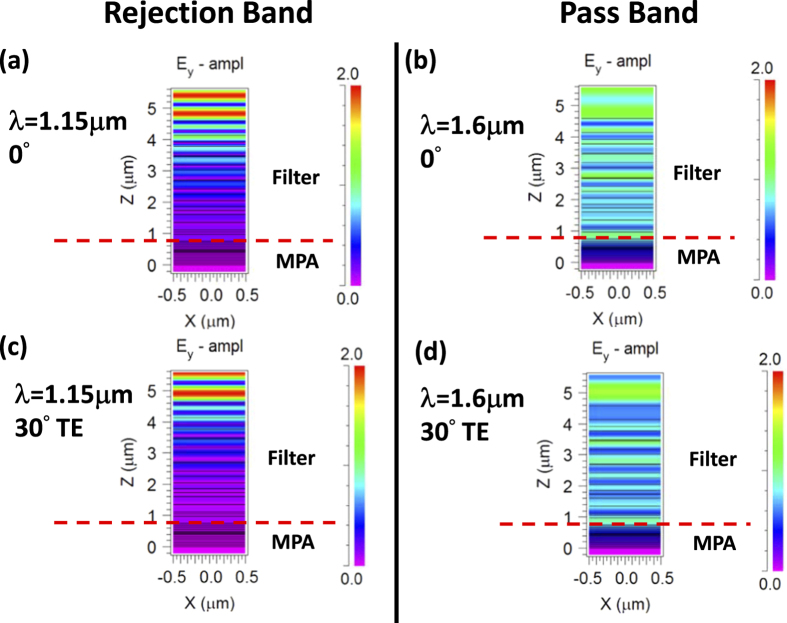
The field profiles for the band-reject (BRJ) MPA. The field profiles of E_y_ amplitude at both normal and oblique incidence are shown for the wavelength in the pass-band and the wavelength in the rejection band, respectively. It can be observed that in the reject band, the photons cannot penetrate the filter, and thus the field profile color is pink in the filter and MPA parts. In the pass band, photons can penetrate the filter, and the field profile color is blue in the filter and gradually change from blue to pink in the MPA indicating the photon absorption.

Figure 4 plots the index profiles to resolve the material layers. Figure 5 plots the field profile of E_y_ amplitude at harmonic steady state for the functionalized MPA band reject (BRJ) case. In the pass-band, it can be seen that the photons penetrate the filter, and when they enter the MPA stack, they are gradually absorbed by the ultrathin Ti layers. In the rejection band, the photons are essentially reflected back by the dielectric filter and never have a chance to reach the MPA stack. In the case of absorption-type filters, the total absorption of the filter and the MPA together is not functionalized, but if only the MPA absorption is concerned such as in sensor applications, the MPA absorption is still fully functionalized and tailored.

## Experiment Result

In this section, we show the experimental result of the band-reject (BRJ) structure. In this case, Si_3_N_4_ and SiO_2_ is chosen to construct the filter part in our structure. Ti and SiO_2_ in the lower part of the structure act as a broadband MPA that shows excellent absorption in the VIS and NIR regimes.

**Figure 6:**
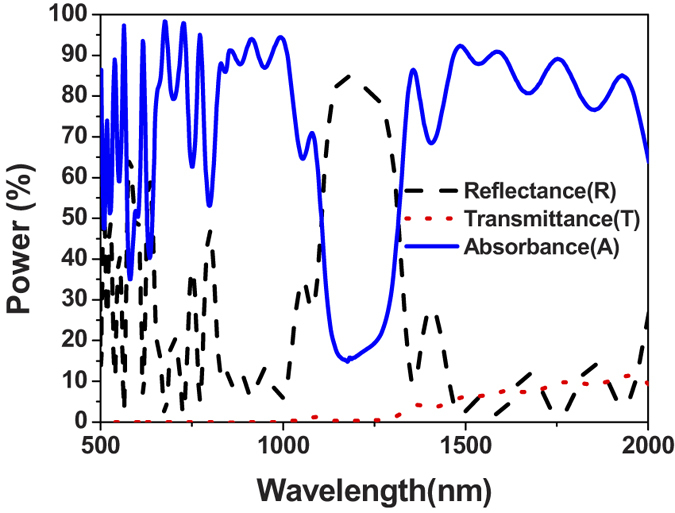
The experiment result of the band-reject (BRJ) structure. Low absorbance at λ = 1000 nm–1250 nm corresponds to the rejection band. Randomly polarized light is used in the measurement. The structure and layer arrangement can be referred to Fig. 2, and Tables 1 and 2.

Both reflectance (R) and transmittance (T) can be measured by ultraviolet visible near-infrared (UV-VIS-NIR) spectroscopy using Hitachi U-4100. Integration sphere is present in this normal incidence measurement setup in Fig. 6. Absorbance (A) can be calculated by 1-Reflectnce (R)-Transmittance (T). Figure 6 shows the experiment result, plotting the reflectance (R), transmittance (T) and absorbance (A). The rejection band in the spectral absorbance is in the range of λ = 1000 nm–1250 nm, verifying the theoretical design in the previous sections. In the passband wavelength ranges, i.e. λ = 600 nm–1000 nm and λ = 1250 nm–2000 nm, high absorbance exists. Therefore, the dielectric filter effectively shapes the MPA absorption spectrum by proper wavelength interference and wave impedance matching. The entire structure, i.e., the MPA plus the dielectric filter, realizes a band-reject functional perfect absorber. Compared to the simulation result of BRJ MPA in Fig. 3, the residual transmittance exists at λ > 1200 nm. The average transmittance λ > 1200 nm is 6.5%. The reason of experimentally measured residual transmittance is due to the slightly lower extinction coefficient (k) of our e-gun deposited Ti, compared to the Rsoft material database values used in the calculation. To circumvent this issue, adjustment in the deposition conditions to tune the Ti extinction coefficients is necessary. Using sputtered Ti films can also be a solution since sputtering provides denser Ti films, which results in higher real and imaginary dielectric constants. Alternatively, a thicker Ti bottom plate or more Ti/SiO_2_ pairs can be deposited to easily resolve the residual transmission issue, but this can increase the total device thickness. Since in our calculation the transmittance is zero, the residual transmittance can certainly be eliminated by adjusting Ti extinction coefficients (k).

**Figure 7:**
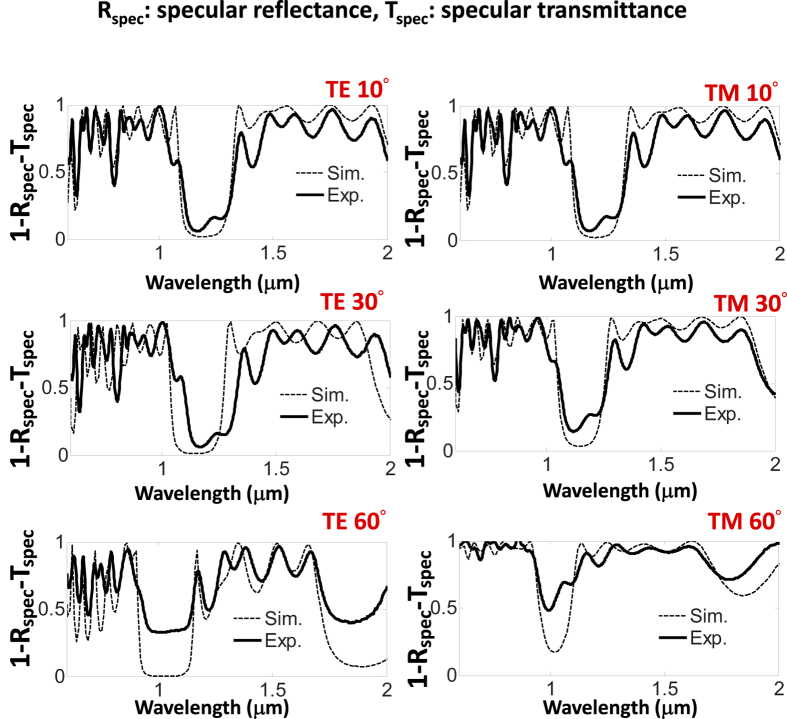
The angular dependence of the band-reject (BRJ) functional absorber spectral characteristics over λ = 1 μm to λ = 2 μm. The BRJ characteristic can sustain until 30°. The dashed line is the simulation result, and the solid line is the experimental result. The specular reflectance (R_spec_) and specular transmittance (T_spec_) are measured by Hitachi variable angle assembly P/N 134-0116. Since the proposed structure is planar, the diffused components of R and T are negligible, and thus 1-R_spec_-T_spec_ is very close to absorbance (A) and also exhibits BRJ characteristics.

The effect of the incident angle and the polarization are investigated in Fig. 7. Figure 7 shows the spectral response measured by Hitachi U-4100 using variable angle assembly Hitachi P/N 134-0116. From Fig. 7, it is demonstrated that the BRJ characteristics of the functional absorbers can sustain until 30°. At 60°, the dielectric filter characteristic begins to degrade, and therefore, the functional absorption also degrades accordingly. The degradation of the dielectric filter at 60° incidence angle can be understood by knowing that the filtering characteristics of planar dielectric multi-layers totally counts on wave interference. The selected layer thicknesses in Table 1 are mainly for normal incidence. At large incidence angle (60°), the optical path lengths inside each dielectric layers are longer compared to the path lengths in the normal incidence case. The phase difference between each reflected/transmitted wave is no longer as designed, and consequently, the wave interference condition that leads to BRJ characteristic breakdowns. A re-designed layer thickness for the SiO_2_/Si_3_N_4_ filter for a specific oblique incidence angle can certainly alleviate this problem. To construct a wide angle functional absorber, a nano-structured filter to enhance the omni-directionality is very effective.

**Figure 8:**
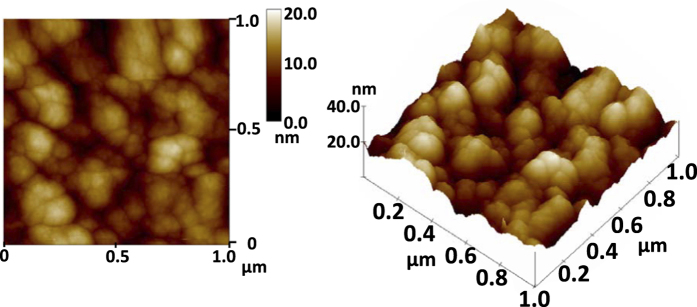
The atomic force microscope (AFM) figure of the band-reject (BRJ) structure. (Left) 2D contour and (right) 3D view. The root mean square roughness is 2.929 nm.

Because our structure consists of many thin layers, surface roughness is an important issue in fabrication. The surface roughness affects the performance of photonic devices, and it should be considered in order to replicate the simulation results reasonably in experiment. Atomic force microscope (AFM) is used to confirm that the surface roughness value in our samples is far below the diffraction limit and does not lead to any far-field effect. Figure 8 shows AFM figure measured by AFM-D3100. The root mean square (RMS) roughness of our device is 2.929 nm. The order of magnitude of this RMS roughness value does not affect the measurement for VIS-NIR range. This is the basis for that the measured response of our fabricated samples can be similar to the simulated ones.

## Discussion

In this work, we propose four kinds of functional absorbers, i.e. low-pass, high-pass, band-reject, and band-pass absorbers. The method we used to achieve a fully functional absorber is using a 10-pair (10X) SiO_2_/Si_3_N_4_ dielectric filter on top of a planar 16-pair (16X) Ti/SiO_2_ metamaterial perfect absorber (MPA). The physics behind the functional absorbers is seemingly quite simple: the filter shapes the spectral response by reflecting the photons with wavelengths in the rejection band. Nevertheless, it is worth to mention that the fact that a filter can be easily integrated with the MPA is totally due to MPA’s planarized nature. A nano-structured configuration can lead to significant near-field diffraction at filter-MPA interface when these two components are integrated together. This complex near-field photon-nanostructure interaction phenomenon leads to imperfect absorption in MPA due to the angular incidence of photons just penetrating the filter. Additionally, the imperfect absorption results in backward diffracted power from the MPA, which can affect the filter characteristic. In our experimental effort, we demonstrate the proposed concepts by a band-reject functional absorber. The rejection band in the range of λ = 1000 nm-1250 nm is very obvious, proving the feasibility of our design. The angular response shows the proposed functional absorber can sustain until 30°. Further increase in incident angle can suffer from the degradation of the dielectric filter. To promote the omnidirectionality, a 2D-filter on a 1D planar MPA can be beneficial. We believe the functional absorbers proposed here can be very useful for future photonic applications.

## Methods

### Theoretical modeling and optimizations

The calculation method in this work is based on rigorously coupled wave analysis (RCWA) and the simulation software used is Rsoft Diffractmod^TM^ 29. Since it is a planar structure, polarization independence is established. Owing to the fact that the wavelengths we are interested in are VIS and NIR, Si_3_N_4_ and SiO_2_ are selected to build the filter, and the titanium (Ti) and SiO_2_ are chosen to construct the broadband metamaterial perfect absorber (MPA). The material parameters are from Rsoft database^29^. Genetic algorithm (GA) is used in this work to locate the appropriate geometry^30^,^31^,^32^,^33^,^34^. GA is a widely used method which imitates evolution rule in biology to tackle the optimization problems. For this reason, GA is selected to calculate the detailed parameters, such as the thickness of each layer and the number of deposited layer necessary to achieve the desired spectral characteristics. We can tailor the absorption band by appropriate design. The aperiodicity in the layer thickness plays an important role to construct an optimized structure. In the GA optimization procedure, 70 generations are implemented, and there are 40 individuals in each generation. The crossover rate is 0.7, the generation gap is 0.9, and the mutation probability is 0.0045. The objective function is the idealized spectral response where the absorbance is one in the pass-band and zero in the rejection band. The transition from the passband to the rejection band is a step function in the idealized spectral responses. For GA with a 40-individual population, the population converges at around 50 generations in our optimization problem. Therefore, here we conduct the GA for 70 generations, and there is no need to define the cut-off condition. To promote the spectral absorption characteristics, several runs may be required to find the desired spectral response. This is because GA is a random process, and some GA runs may converge to moderate spectral responses. The band-pass (BP) layer thickness is directly from literature^35^.

### Sample fabrication

Ti films and SiO_2_ films are deposited on the silicon wafer alternatively by electron-gun (e-gun) evaporator AST PEVA 600I. The pressure in the process is kept <3 × 10^−6^ torr at Ti deposition and <5 × 10^−6^ torr at SiO_2_ deposition. Firstly, a Ti layer with a thickness of 200 nm is deposited on the bare silicon wafer. Afterward, 44.3 nm SiO_2_ and 2.3 nm Ti are deposited on the Ti bottom layer. 16 pairs of SiO_2_/Ti alternating layer are deposited sequentially. After the MPA deposition, plasma-enhanced chemical vapor deposition (PECVD) is used for the deposition of the dielectric filter, which is on top of the MPA. SiO_2_ layers are deposited with precursors SiH_4_ and N_2_O at 300 °C and 100 mtorr. Si_3_N_4_ layers are deposited with precursors SiH_4_ and NH_3_ with the same condition. Repeated deposition of SiO_2_ films and Si_3_N_4_ films is conducted until 10 pairs of SiO_2_/Si_3_N_4_ alternating aperiodic layers are finished. The thickness of each layer is different achieving an effective aperiodic filter.

### Sample measurement

**Figure 9:**
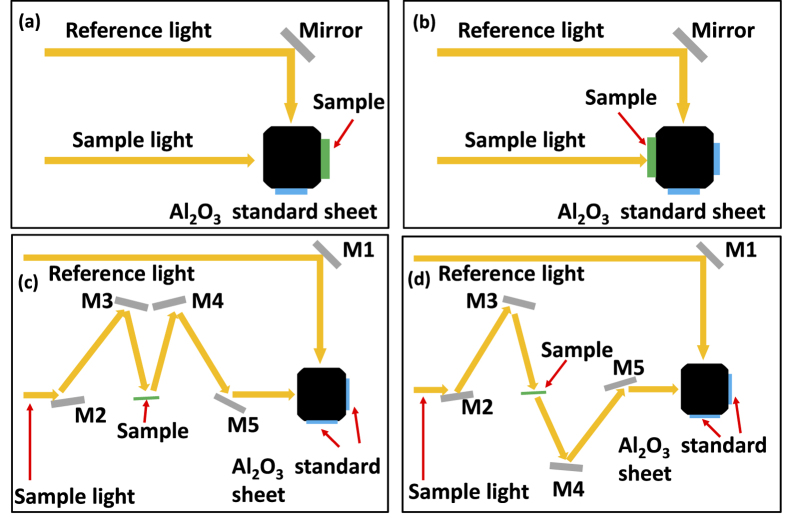
The illustration of the UV-VIS-NIR reflectance and transmittance measurement setup. (**a**) The measurement setup for the reflectance (R) at normal incidence. (**b**) The measurement setup for the transmittance (T) at normal incidence. (**c**) The measurement setup for the specular reflectance (R_spec_) at oblique incidence. (**d**) The measurement setup for the specular transmittance (T_spec_) at oblique incidence.

The reflectance (R) and transmittance (T) in Fig. 6 are measured by ultraviolet visible near-infrared (UV-VIS-NIR) spectroscopy using Hitachi U-4100 with built-in integration sphere, illustrated in Fig. 9(a,b). Absorbance (A) can be calculated by 1-Reflectnce (R)-Transmittance (T). The specular reflectance (R_spec_) and specular transmittance (T_spec_) at oblique incidence angles in Fig. 7 are measured by Hitanchi U-4100 with Hitachi variable angle assembly P/N 134-0116, illustrated in Fig. 9(c,d). The surface roughness data in Fig. 8 is measured by Digital Instrument^TM^ Dimension 3100 AFM using tapping mode.

## Additional Information

**How to cite this article**: Fu, S. M. *et al*. A fully functionalized metamaterial perfect absorber with simple design and implementation. *Sci. Rep.* **6**, 36244; doi: 10.1038/srep36244 (2016).

**Publisher’s note**: Springer Nature remains neutral with regard to jurisdictional claims in published maps and institutional affiliations.

## Figures and Tables

**Figure 1:**
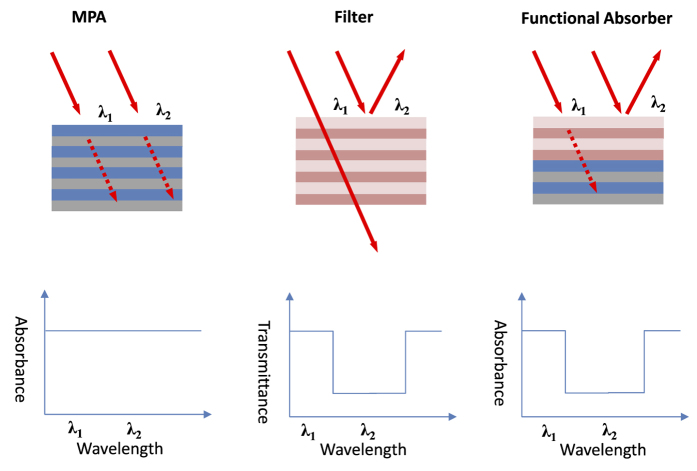
Illustration of the concepts of the proposed design in this work. Originally we have a uniform broadband MPA where photons of all wavelengths are absorbed. For a standalone filter, λ_1_ represents the wave that can pass the filter, and λ_2_ represents the wave that is rejected. After the filter is integrated with the MPA, λ_1_ is absorbed while λ_2_ is not. The perfect absorption of the planar MPA eliminates the backward reflection between the filter-MPA interface. This is the key to maintaining the filter characteristics to be the same as its standalone one.

**Table 1:** The dielectric filter layer thicknesses.

Functional MPA type	L1	L2	L3	L4	L5	L6	L7
LP	179	168	438	168	291	249	272
HP	48	71	114	457	83	133	71
BP	0	172	178	173	205	173	205
BRJ	226	71	71	241	241	129	133
Functional MPA type	L8	L9	L10	L11	L12	L13	L14
LP	203	295	199	303	496	264	176
HP	152	79	91	141	64	102	388
BP	173	205	173	205	173	205	159
BRJ	187	206	164	260	29	310	152
Functional MPA type	L15	L16	L17	L18	L19	L20	L21
LP	230	172	218	149	199	172	—
HP	98	121	67	214	121	60	—
BP	231	148	178	148	178	148	178
BRJ	272	44	276	141	233	438	—
Functional MPA type	L22	L23	L24	L25	L26	L27	L28
LP	—	—	—	—	—	—	—
HP	—	—	—	—	—	—	—
BP	148	178	148	245	126	198	0
BRJ	—	—	—	—	—	—	—

Thickness is in nanometer (nm)^a^.

^a^The order of the list is from the bottom layer to the topmost layer. Alternating SiO_2_/Si_3_N_4_ layers are deposited. L1 is SiO_2_, L2 is Si_3_N_4_, L3 is SiO_2_, etc. In the case of BP functional absorbers, Si_3_N_4_ is replaced with Si.

**Table 2:** The MPA metal and dielectric spacer thickness.

Functional MPA type	LP	HP	BRJ	BP
t_metal_	1.4	1.6	2.3	2.0
t_oxide_	8	40	44	80

Thickness is in nanometer (nm).
